# Long Noncoding RNA SNHG12 Promotes Gastric Cancer Proliferation by Binding to HuR and Stabilizing YWHAZ Expression Through the AKT/GSK-3β Pathway

**DOI:** 10.3389/fonc.2021.645832

**Published:** 2021-06-14

**Authors:** Tianqi Zhang, Maneesh Kumarsing Beeharry, Yanan Zheng, Zhenqiang Wang, Jianfang Li, Zhenggang Zhu, Chen Li

**Affiliations:** Department of General Surgery, Shanghai Key Laboratory of Gastric Neoplasms, Shanghai Institute of Digestive Surgery, Ruijin Hospital, Shanghai Jiao Tong University School of Medicine, Shanghai, China

**Keywords:** gastric cancer, proliferation, SNHG12, HuR, YWHAZ

## Abstract

**Background:**

Gastric cancer (GC) is a malignancy with high morbidity and mortality rates worldwide. SNHG12 is a long noncoding RNA (lncRNA) commonly involved many types of cancers in the contexts of tumorigenesis, migration and drug resistance. Nevertheless, its role in GC proliferation is poorly understood.

**Methods:**

Bioinformatics and qRT-PCR assays were used to analyze the expression of SNHG12 in GC tissues and cells. *In vitro* and *in vivo* experiments were conducted to detect the role of SNHG12 in GC development. qRT-PCR, PCR, western blotting (WB), RNA binding protein immunoprecipitation (RIP), immunoprecipitation (IP), immunohistochemistry (IHC), fluorescence *in situ* hybridization (FISH) and *in situ* hybridization (ISH) were performed to investigate the underlying mechanisms by which SNHG12 promotes GC proliferation.

**Results:**

SNHG12 was highly expressed in GC cells and tissues, and predicted poor survival. *In vitro* and *in vivo* assays showed that SNHG12 knockdown inhibited GC proliferation, while SNHG12 overexpression promoted GC proliferation. Further experiments confirmed that SNHG12 was mainly located in the cytoplasm and bound to HuR. Bioinformatics analysis predicted that YWHAZ was the common target of SNHG12 and HuR, and that the “SNHG12-HuR” complex enhanced the stability of YWHAZ mRNA. Furthermore, YWHAZ, which was highly expressed in GC, predicted poor survival and promoted GC proliferation by phosphorylating AKT. Rescue assays verified that SNHG12 promoted GC proliferation by activating the AKT/GSK-3β pathway.

**Conclusions:**

SNHG12 binds to HuR and stabilizes YWHAZ. SNHG12 promotes GC proliferation *via* modulation of the YWHAZ/AKT/GSK-3β axis *in vitro* and *in vivo*. Thus, SNHG12 could become a novel therapeutic target for anti-tumor therapy.

## Introduction

Gastric cancer (GC) is a malignancy that can easily invade and proliferate in adjacent regions and poses a serious threat to human health worldwide (1). Although neoadjuvant and systemic radio-chemotherapy have shown some benefits for the management of GC, its prognosis remains disappointing due to metastasis and recurrence (2, 3). The identification of biomarkers and their underlying mechanisms associated with GC tumorigenesis and proliferation show promise for facilitating early diagnosis and prompting precision therapy (4).

Long noncoding RNAs (lncRNAs) are evolutionarily conserved RNA molecules with a length of more than 200 nucleotides that lack protein-coding ability (5). Numerous studies indicate that lncRNAs play various functional roles in multiple kinds of biological processes, including cell growth, invasion, migration, and tumorigenesis (6, 7). The oncogenic role of lncRNA small nucleolar RNA host gene 12 (SNHG12) has been verified in recent studies (8). LncRNA SNHG12 promotes temozolomide resistance in glioblastoma (9). In renal cell carcinoma, SNHG12 promotes proliferation, migration, invasion and sunitinib resistance *via* the SNHG12/SP1/CDCA3 axis (10). In addition, previous studies also reported that SNHG12 promotes GC proliferation, migration by sponging miR-320 and miR-16 (11, 12). However, the possible interactions between SNHG12 and other genes and signaling pathways in GC remains to be elucidated.

LncRNAs stimulate the pathogenesis of GC through their participation in key signaling pathways: for example, linc00662 promotes colon cancer tumor growth and metastasis by activating the ERK signaling pathway (13), and lncRNA PSTAR inhibits hepatic carcinoma cell proliferation and tumorigenesis by inducing p53-mediated cell cycle arrest (14). Herein, we found that when its expression was increased, SNHG12 reduced the overall survival of GC patients and promoted GC tumorigenesis by activating the AKT/GSK-3β pathway and binding with the RNA binding protein ELAVL1 (also known as HuR) and stabilizing YWHAZ expression. Thus, SNHG12 shows promise as a biomarker for prognosis prediction and a possible therapeutic target in GC patients.

## Materials and Methods

### GC Patients and Tissue Specimens

A total of 16 GC tissues and the corresponding adjacent non-cancerous epithelial tissues were obtained from GC patients undergoing radical surgery from March to July 2020 at Ruijin Hospital affiliated with Shanghai Jiao Tong University School of Medicine. The patients did not undergo radiotherapy or chemotherapy prior to surgery. All cases were independently diagnosed histologically by two experienced pathologists and staged according to the tumor-node-metastasis (TNM) staging system of the American Joint Committee on Cancer (AJCC 7th ed., 2010). All tissue samples were immediately frozen in liquid nitrogen after resection from patients and stored at −80°C for further analysis. The acquisition of the tissues was approved by the Ruijin Hospital Ethics Committee.

### Cell Culture

GC cell lines (MGC-803, AGS, HGC-27, MKN-28, MKN-45, SGC-7901, BGC-823) and the non-malignant gastric mucosal epithelial cell line (GES-1) were purchased from the Cell Bank of the Chinese Academy of Sciences (Shanghai, China). DMEM (Meilunbio, #MA0212-1) with 10% newborn calf serum (Bioagrio, #S1105-100) was used for cell culture (37°C in 5% CO_2_).

### RNA Extraction, Quantitative Reverse Transcription PCR (qRT-PCR) and PCR

Total RNA was extracted from cultured cells and tissues using TRIzol reagent (Vazyme, #R401-01) according to the manufacturer’s instructions. A cytoplasmic and nuclear RNA purification kit (#21000, 37400) was purchased from NORGEN. RNA was reverse transcribed into cDNA using HiScript III RT SuperMix for qPCR (Vazyme, #R323-01). cDNA was quantified by qRT-PCR and the data were acquired with SYBR Green (Vazyme, #Q711-02/03) using an Applied Biosystems 7500 instrument. Taq Master Mix (Dye Plus) (Vazyme, #P112-01) was used in PCR. GAPDH and ACTB were used as internal controls. Primers are listed in **Supplementary 1**.

### Lentivirus Production, siRNA, Plasmids and Cell Transfection

Lentivirus-containing short hairpin RNA (shRNA) targeting SNHG12 was purchased from OBiO (Shanghai, China), and the pCDH-CMV-Human vector for SNHG12 overexpression was purchased from Allwin (Shanghai, China). SiRNAs for YWHAZ, HuR and negative control (NC) oligonucleotides were obtained from Sangon Biotech (Shanghai, China). GC cells were transfected with the above-mentioned oligonucleotides and plasmids using Lipofectamine 2000 (Invitrogen, #1875894) according to the manufacturer’s protocol. The sequences of the siRNAs are listed in **Supplementary 1**.

### Western Blotting

Total proteins from cells were extracted using RIPA buffer supplemented with protease inhibitors and phosphatase inhibitors. Primary antibodies against YWHAZ (ABclonal, #A13370), GAPDH (Proteintech, #60004-1-Ig), AKT (Cell Signaling Technology, #4691S), p-AKT (Cell Signaling Technology, #13038S), GSK-3β (Cell Signaling Technology, #9315S), p-GSK-3β (ABclonal, #AP1088), and ELAVL1 (Cell Signaling Technology, #12582S) were used in this study.

### Cell Proliferation Detection

For the Cell Counting Kit-8 (CCK-8) assay, 100 μl of cell suspension with 3,000 cells was seeded into each well of a 96-well plate. Every day, 10 μl of CCK-8 solution was added to each well and cultured for 2.5 h. Then, the absorbance at 450 nm was measured by a microplate reader (BioTek Instruments). For colony formation assays, 1,500 cells per well were seeded into six-well plates and cultured for 10 days. Colonies were fixed and stained with 0.5% crystal violet. EdU assays were conducted according to the protocol of the Cell-Light EdU Apollo567 In Vitro Kit (#C10310-1), which was purchased from RiboBio company. Cells were cultured in 24-well plates, and 50 μM EdU labeling medium was added to the cells for 2 h the next day. The cells were then treated with 4% paraformaldehyde (pH 7.4) for 30 min and then 0.5% Triton X-100 for 20 min at room temperature. The samples were stained with anti-EdU working solution and subsequently incubated with Hoechst 33342 (5 μg/ml). The percentage of EdU-positive cells was measured under fluorescence microscopy. Four fields of view were randomly selected in each well to determine the percentage of EdU-positive cells.

### RNA Binding Protein Immunoprecipitation

RNA binding protein immunoprecipitation (RIP) was performed using the EZ-Magna RIP Kit (Millipore 17-700) according to the manufacturer’s protocol, and the antibody used in this assay was ELAVL1 (Cell Signaling Technology, #12582S). The primers used in this assay are listed in **Supplementary 1**.

### Fluorescence *In Situ* Hybridization (FISH) and *In Situ* Hybridization (ISH)

The FISH assays of GC cells and ISH assays of tissues were conducted according to the method described as previously (15, 16).

### Immunohistochemistry (IHC)

IHC staining was performed as previously described (15). Primary anti-bodies against YWHAZ (ABclonal, #A13370), AKT (Cell Signaling Technology, #4691S), p-AKT (Cell Signaling Technology, #13038S), GSK-3β (Cell Signaling Technology, #9315S), and p-GSK-3β (ABclonal, #AP1088) were used in this study.

### *In Vivo* Animal Assays

Four-week-old immunodeficient BALB/c female nude mice were randomly divided into two groups (n = six for each group). MGC-803 cells (2 × 10^7^) with stable sh-SNHG12 or empty vector were separately subcutaneously injected into the flanks of the subjects. After 1 month, the mice were sacrificed, and the tumors were dissected for volume measurement and further immunohistochemical investigations.

### Statistical Analysis

All statistical analyses were conducted using SPSS 23.0 (SPSS, Chicago, IL, USA) or GraphPad Prism V8 (GraphPad Prism, Inc., La Jolla, CA, USA). Each experiment was performed at least in triplicate, and data are presented as the mean ± SD of three independent experiments. Student’s t-test or one-way ANOVA was used to compare the means of two or three groups. Differences with P values less than 0.05 were considered statistically significant.

## Results

### SNHG12 Is Overexpressed in GC and Predicts a Poor Prognosis

**Figure 1:**
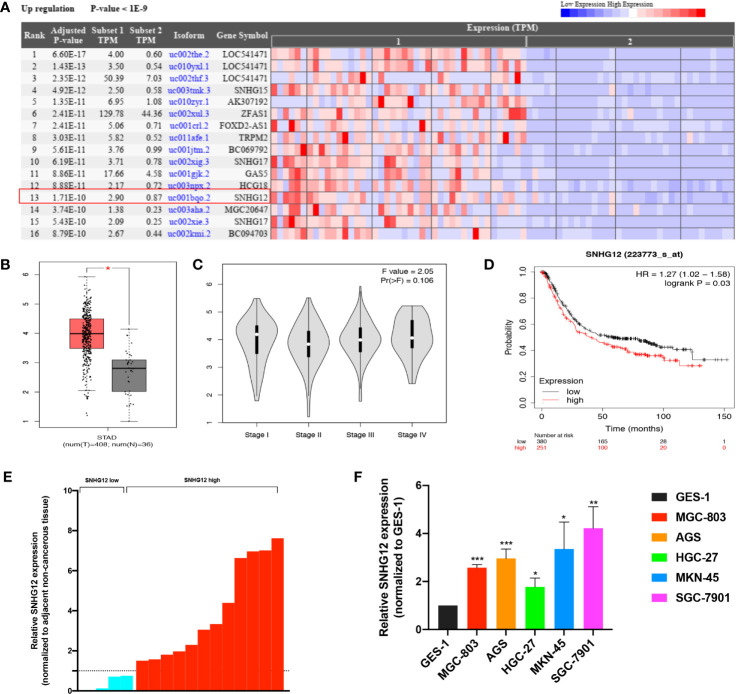
SNHG12 is highly expressed in GC tissues and cells and predicts poor survival. **(A)** Upregulated lncRNAs in GC tissues according to the Cancer RNA-Seq Nexus online tool (http://syslab4.nchu.edu.tw/). **(B)** SNHG12 expression in GC tissues compared with matched normal epithelial tissues according to the GEPIA online tool (http://gepia.cancer-pku.cn/index.html). **(C)** SNHG12 expression in various stages of GC in GEPIA. **(D)** Survival analysis of SNHG12 using the Kaplan–Meier plotter online tool (https://kmplot.com/analysis/). **(E)** SNHG12 expression in GC tissues compared with adjacent non-cancerous tissues by qRT-PCR. **(F)** SNHG12 expression in GC cell lines detected by qRT-PCR. Significant results are presented as *P < 0.05, **P < 0.01, ***P < 0.001.

Based on bioinformatic database analysis, we explored the relationship between dysregulated lncRNAs and GC development. The Cancer RNA-Seq Nexus online tool (http://syslab4.nchu.edu.tw/) was used to analyze the upregulated lncRNAs in GC tissues (**Figure 1A**). Among them, SNHG12 was significantly highly expressed in GC tissues. According to the Gene Expression Profiling Interactive Analysis (GEPIA) online tool (http://gepia.cancer-pku.cn/index.html), SNHG12 expression was higher in tumor tissues from patients with various stages of GC than in matched normal tissues in the stomach adenocarcinoma (STAD) dataset (**Figures 1B, C** **)**. In the Kaplan–Meier plotter online tool (https://kmplot.com/analysis/), high SNHG12 expression was shown to indicate poor survival in GC (**Figure 1D**). Hence, we conducted SNHG12 expression profiling in GC tissues and GC cell lines. SNHG12 was significantly highly expressed in GC tissues compared with the corresponding adjacent non-cancerous epithelial tissues (**Figure 1E**). On the other hand, SNHG12 was highly upregulated in all GC cell lines assessed, and we chose MGC-803 and AGS cells for further assays (**Figure 1F**).

### SNHG12 Promotes GC Cells Proliferation

**Figure 2:**
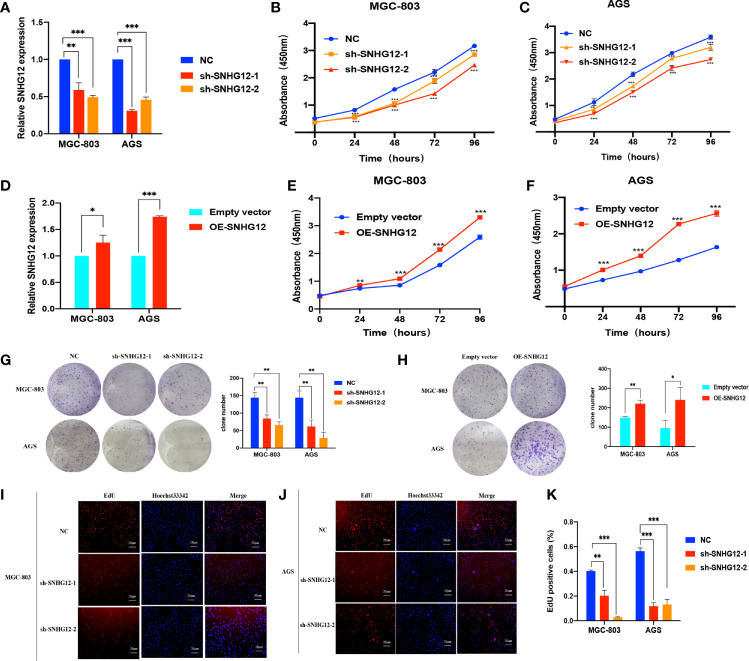
SNHG12 promotes GC cells proliferation. **(A)** Efficiencies of SNHG12 knockdown by qRT-PCR. **(B, C)** CCK-8 assays showing the effects of SNHG12 knockdown on cell proliferation. **(D)** Efficiencies of SNHG12 overexpression by qRT-PCR. **(E, F)** CCK-8 assays showing the effects of SNHG12 overexpression on cell proliferation. **(G, H)** Colony formation assays showing the role of SNHG12 on GC cell proliferation. **(I–K)** EdU assays showing the regulation of SNHG12 on GC cell proliferation. Significant results are presented as *P < 0.05, **P < 0.01, ***P < 0.001.

To verify the role of SNHG12 in GC proliferation, gain and loss assays were conducted in MGC-803 and AGS cells: shRNA vectors were used to knockdown SNHG12, and compared with the NC, the expression levels in sh-SNHG12-1 or sh-SNHG12-2 were both significantly inhibited in MGC-803 and AGS cells (p <0.01) (**Figure 2A**). As shown in **Figures 2B, C**, the results of CCK-8 assays demonstrated that MGC-803 and AGS cells transfected with the sh-SNHG12-1 and sh-SNHG12-2 vectors proliferated more slowly than those transfected with the NC vectors (p <0.01). On the other hand, to overexpress SNHG12, pCDH-CMV-human vectors were transfected into MGC-803 and AGS cells. **Figure 2D** shows that SNHG12 expression in MGC-803 cells transfected with pCDH-CMV-human vectors was 1.25-fold higher than that in the NC cells, while in AGS cells, it was approximately 1.7-fold higher (both p <0.05). CCK-8 assays showed that upon SNHG12 overexpression, MGC-803 and AGS cells proliferated faster than the NC cells (p <0.01) (**Figures 2E, F** **)**. Colony formation assays were conducted to elucidate the effect of SNHG12 on GC cell proliferation. Upon SNHG12 knockdown, the colony numbers of MGC-803 cells decreased by approximately 40% in the sh-SNHG12-1 group or 60% in the sh-SNHG12-2 group compared with the NC, while in AGS cells, the colony numbers decreased by approximately 60% in the sh-SNHG12-1 group or 80% in the sh-SNHG12-2 group (all p <0.05) (**Figure 2G**). On the other hand, upon SNHG12 overexpression, the colony numbers of MGC-803 and AGS cells were 1.5-and 2.5-fold higher, respectively, than that of the NC cells (all p <0.05) (**Figure 2H**). EdU assays showed that the proportion of Edu-positive MGC-803 cells decreased by 50% in the sh-SNHG12-1 group and 92.5% in the sh-SNHG12-2 group, while that of AGS cells decreased by 78% in the sh-SNHG12-1 group and 77% in the sh-SNHG12-2 group upon SNHG12 suppression (all p <0.01) (**Figures 2I–K** **)**. The above results indicated that SNHG12 indeed promotes GC cell proliferation.

### SNHG12 Binds to HuR and Stabilizes mRNA ELAVL1

**Figure 3:**
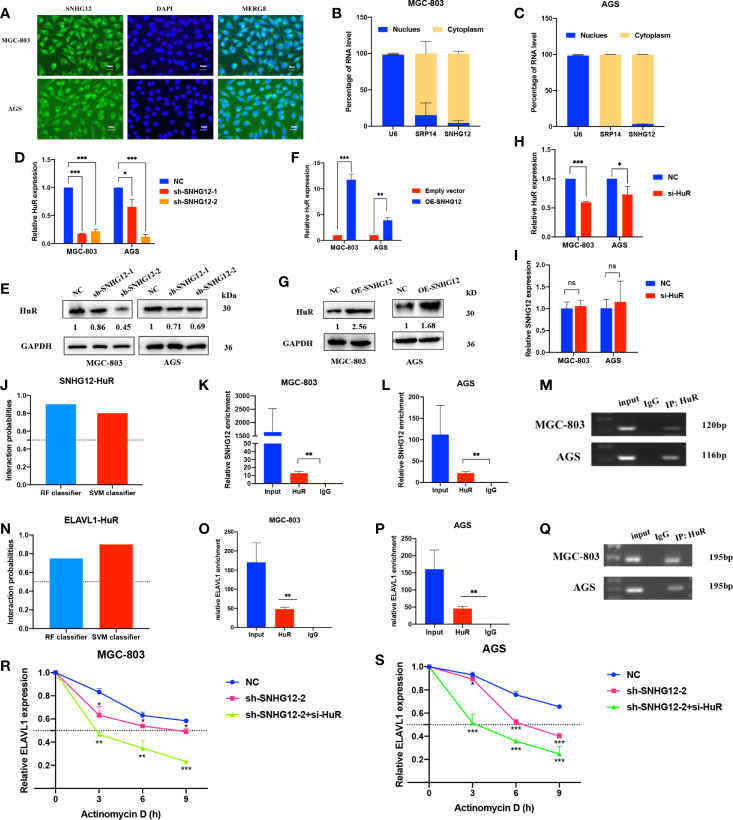
SNHG12 binds to HuR and stabilizes ELAVL1 mRNA. **(A–C)** FISH assays and cytoplasmic and nuclear RNA purification assays indicate that SNHG12 is mainly located in the GC cell cytoplasm. **(D–G)** qRT-PCR and WB assays showing the expression of HuR at the RNA and protein levels upon SNHG12 knockdown and overexpression. Numbers show the quantification of the relative protein amount. **(H, I)** qRT-PCR assays showing the efficiency of HuR knockdown and its effect on the regulation of SNHG12. **(J)** Prediction of the interaction probabilities of SNHG12 and HuR by bioinformatics (http://pridb.gdcb.iastate.edu/RPISeq/). Predictions with probabilities >0.5 were considered as “positive”, indicating that the RNA more likely to interact with the protein than not to interact. **(K–M)** RIP assays showing that SNHG12 binds to HuR. **(N)** Prediction of the interaction probabilities of ELAVL1 and HuR by bioinformatics (http://pridb.gdcb.iastate.edu/RPISeq/). **(O–Q)** RIP assays showing that ELAVL1 binds to HuR. **(R, S)** RNA stability assays were conducted using actinomycin D to disrupt RNA synthesis in MGC-803 and AGS cells, and the degradation rates of ELAVL1 mRNAs were tested every 3 h. Significant results are presented as ns P > 0.05, *P < 0.05, **P < 0.01, ***P < 0.001. Magnification ×400; scale bar, 20 μm.

To elucidate the underlying mechanisms by which SNHG12 regulates GC proliferation, it is important to first confirm the intracellular location of SNHG12. FISH assays showed that SNHG12 was mainly located in the cytoplasm (**Figure 3A**), and this finding was also supported by cytoplasmic and nuclear RNA purification assays (**Figures 3B, C** **)**. These findings suggested that SNHG12 mainly exerted its function at the post- transcriptional level and might cooperate with RNA binding proteins. HuR, a popular RNA binding protein (RBP) encoded by ELAVL1, can enhance the stability of mRNAs. qRT-PCR and western blotting (WB) assays showed that upon knockdown or overexpression of SNHG12, the relative HuR expression at the RNA and protein levels decreased or increased, respectively (**Figures 3D–G** **)**. However, HuR knockdown did not change the expression of SNHG12 (**Figures 3H, I** **)**. We further used bioinformatics (http://pridb.gdcb.iastate.edu/RPISeq/) to predict the interaction between SNHG12 and HuR, and the random forest (RF) classifier and support-vector machine (SVM) classifier scores were 0.9 and 0.8, respectively (**Figure 3J**), suggesting that SNHG12 has a high probability of binding to HuR. RIP assays revealed that SNHG12 bound to HuR in MGC-803 and AGS cells (**Figures 3K–M** **)**. To further investigate the mechanisms by which SNHG12 regulates HuR, we investigated whether ELAVL1 mRNA could bind to HuR. The RNA–Protein Interaction Prediction (RPISeq) online tool predicted the interaction between ELAVL1 and HuR: the RF classifier and SVM classifier scores were 0.75 and 0.9, respectively (**Figure 3N**). RIP assays verified the prediction that ELAVL1 could bind to HuR (**Figures 3O–Q** **)**. Furthermore, to verify whether the SNHG12-HuR complex could enhance the stability of ELAVL1 mRNA, MGC-803 and AGS cells were transfected with NC shRNA, sh-SNHG12-2 or sh-SNHG12-2 and si-HuR and then treated with actinomycin D, which can inhibit RNA synthesis. Cells were harvested every 3 h to obtain RNA for qRT-PCR assays. The results showed that the half-life of ELAVL1 mRNA was significantly reduced in the SNHG12 and HuR knockdown groups compared with the NC group (**Figures 3R, S**).

### SNHG12 Enhances the Stability of YWHAZ by Binding to HuR

**Figure 4:**
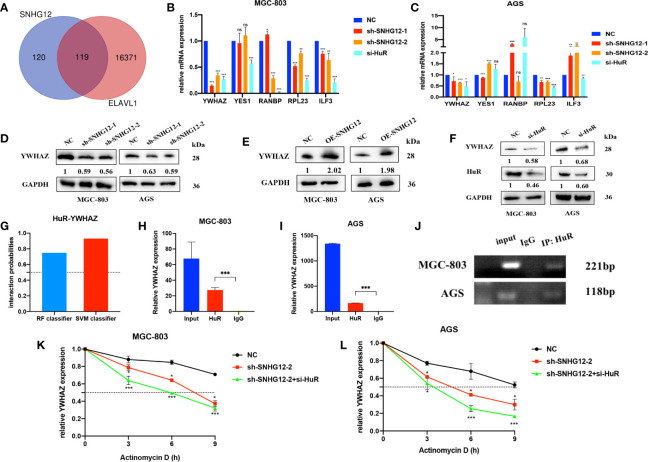
The SNHG12-HuR complex enhances the stability of YWHAZ. **(A)** mRNAs positively correlated with SNHG12 and HuR according to StarBase. **(B, C)** qRT-PCR assays showing the changes in several mRNAs involved in cell proliferation upon SNHG12 or HuR knockdown. **(D–F)** WB assays showing the expression of YWHAZ protein levels upon SNHG12 and HuR knockdown. Numbers show the quantification of the relative protein amount. **(G)** Prediction of the interaction probabilities of YWHAZ and HuR by bioinformatics (http://pridb.gdcb.iastate.edu/RPISeq/). Predictions with probabilities >0.5 were considered as positive, indicating that the RNA was more likely to interact with the protein than to not interact. **(H–J)** RIP assays showing that YWHAZ binds to HuR. **(K, L)** RNA stability assays showing the degradation rates of YWHAZ mRNAs tested every 3 h. Significant results are presented as ns P > 0.05, *P < 0.05, **P < 0.01, ***P < 0.001.

We used the StarBase online tool to collecte mRNAs positively correlated with SNHG12 and ELAVL1 and overlapped the results (**Figure 4A** and **Supplementary 2**). Among the 119 common genes, we chose some mRNAs that were highly positively correlated with SNHG12 and ELAVL1 and associated with cell proliferation: YWHAZ, YES1, RNABP, RPL23 and ILF3. Upon SNHG12 or HuR knockdown, YWHAZ expression decreased the most, as detected by qRT-PCR (**Figures 4B, C** **)**. After comprehensive consideration, we chose YWHAZ as the research target. YWHAZ, encodes the 14-3-3ζ protein, which is a well-known protein involved in many signal transduction and tumor progression (17). WB assays illustrated that the YWHAZ protein was positively related to SNHG12 and HuR (**Figures 4D–F** **)**. Furthermore, we used bioinformatics to predict the interaction between YWHAZ and HuR, and the RF classifier and SVM classifier scores were 0.75 and 0.93, respectively (**Figure 4G**). Then, RIP assays were conducted, and the results showed that YWHAZ could bind to HuR in MGC-803 and AGS cells (**Figures 4H–J** **)**. Then, RNA stability assays were conducted to verify whether the SNHG12–HuR complex could enhance the stability of YWHAZ mRNAs. The results showed that the half-life of YWHAZ mRNA was significantly reduced in the SNHG12 and HuR knockdown groups compared with the NC group (**Figures 4K, L** **)**. In conclusion, the SNHG12–HuR complex can regulate the stability of YWHAZ.

### YWHAZ Promotes GC Cell Proliferation *Via* the AKT/GSK-3β Pathway

**Figure 5:**
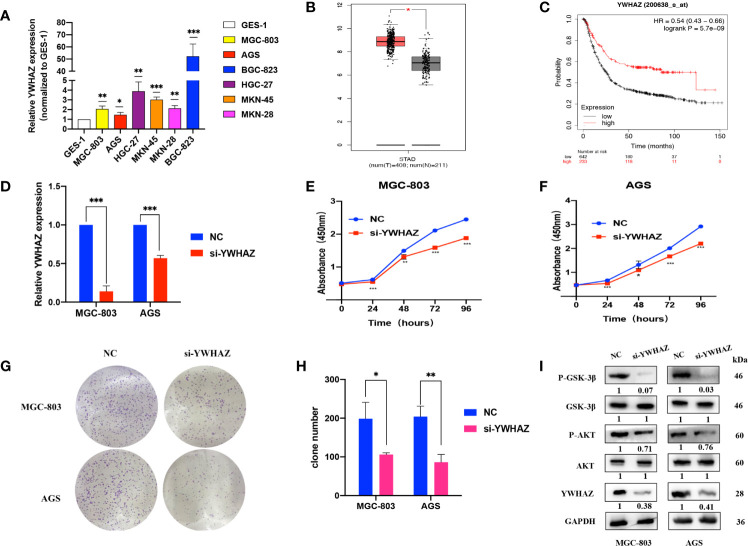
YWHAZ is highly expressed in GC and promotes GC cell proliferation. **(A)** qRT-PCR assays showing YWHAZ expression in GC cells normalized to GES-1. **(B)** YWHAZ expression in GC tissues from the GEPIA online tool (http://gepia.cancer-pku.cn/index.html). **(C)** Survival analysis of YWHAZ using the Kaplan–Meier plotter online tool (https://kmplot.com/analysis/). **(D)** Efficiencies of YWHAZ knockdown in MGC-803 and AGS cells by qRT-PCR. **(E, F)** CCK-8 assays showing the effects of YWHAZ on GC cell proliferation. **(G, H)** Colony formation assays showing the potential of YWHAZ to affect GC cell proliferation. **(I)** WB assays showing the effect of YWHAZ on the AKT/GSK-3β pathway. Numbers show the quantification of the relative protein amount. Significant results are presented as *P < 0.05, **P < 0.01, ***P < 0.001.

qRT-PCR assays showed that YWHAZ was upregulated in GC cell lines compared to GES-1 cells (**Figure 5A**). We used the GEPIA online tool (http://gepia.cancer-pku.cn/index.html) and found that YWHAZ expression was high in tumor tissues (**Figure 5B**). Kaplan–Meier survival analysis using the Kaplan–Meier plotter online tool (https://kmplot.com/analysis/) indicated that patients with high YWHAZ expression had poor outcomes (**Figure 5C**). Upon transfection of si-YWHAZ, the efficiencies of YWHAZ knockdown were over 30% in MGC-803 and AGS cells; CCK-8 assays showed that proliferation of GC cells was slower in the si-YWHAZ groups than in the NC groups; colony formation assays indicated that the colony numbers for both MGC-803 and AGS cells decreased by approximately 50% in the si-YWHAZ groups compared with the NC group (all p <0.05) (**Figures 5D–H** **)**. Previous studies demonstrated that YWHAZ could promote glioma cell invasion by activating the PI3K/AKT pathway (18). Thus, WB assays showed that the protein expression levels of YWHAZ, p-AKT, and p-GSK-3β were decreased upon YWHAZ knockdown, while the total expression of AKT and GSK-3β did not change in MGC-803 and AGS cells (**Figure 5I**).

### SNHG12 Promotes GC Cell Proliferation *Via* the YWHAZ/AKT/GSK-3β Axis

**Figure 6:**
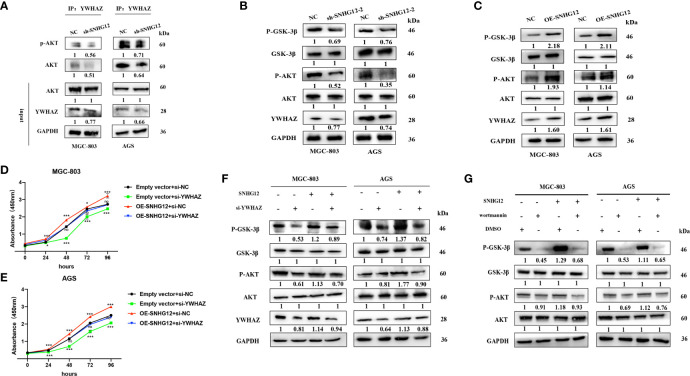
SNHG12 promotes GC proliferation *via* the AKT/GSK-3β pathway. **(A)** IP assays showing that YWHAZ interacts with and phosphorylates AKT. **(B, C)** WB assays showing the effect of SNHG12 on the AKT/GSK-3β pathway. **(D–F)** Rescue assays validated that SNHG12 promotes GC cell proliferation by regulating YWHAZ *via* the AKT/GSK-3β pathway. **(G)** SNHG12 activates AKT pathway. Numbers show the quantification of the relative protein amount. Significant results are presented as *P < 0.05, ***P < 0.001.

It has been previously reported that YWHAZ can mediate signal transduction by binding to phosphoserine-containing proteins (19). To further investigate its role in the AKT pathway, IP assays were conducted, and the results showed that compared with that in the NC groups, AKT and p-AKT expression was enriched in the si-YWHAZ group but decreased in the sh-SNHG12 groups, while total AKT protein expression in the NC and sh-SNHG12 groups was not different (**Figure 6A**). In addition, SNHG12 knockdown led to decreased expression of YWHAZ, p-AKT, and p-GSK-3β, but the expression of AKT and GSK-3β did not change (**Figure 6B**). Moreover, overexpression of SNHG12 resulted in increased expression of YWHAZ, p-AKT, and p-GSK-3β, and the expression of AKT and GSK-3β did not change (**Figure 6C**). Then, we conducted rescue assays, and transfected MGC-803 and AGS cells with NC siRNA, si-YWHAZ, pcDNA-SNHG12, or pcDNA-SNHG12 plus si-YWHAZ. CCK-8 assays showed that YWHAZ knockdown suppressed GC cell proliferation and that SNHG12 overexpression promoted GC cell proliferation. On the other hand, YWHAZ knockdown and SNHG12 overexpression resulted in no differencein GC proliferation compared to the NC groups (**Figures 6D, E** **)**. WB assays showed that the expression of YWHAZ, p-AKT, and p-GSK-3β decreased upon YWHAZ knockdown; while the expression of YWHAZ, p-AKT, and p-GSK-3β increased upon SNHG12 overexpression. Furthermore, when YWHAZ was knocked down and SNHG12 was overexpressed simultaneously, the expression of YWHAZ, p-AKT, and p-GSK-3β was rescued compared to knockdown YWHAZ or overexpression SNHG12. However, the total protein levels of AKT and GSK-3β did not change (**Figure 6F**).

To further investigate whether SNHG12-mediated GC proliferation depended on the activation of the AKT pathway, we treated MGC-803 and AGS cells with wortmannin, a specific PI3K inhibitor. SNHG12 significantly elevated the protein levels of p-AKT and p-GSK-3β, and the effects were reversed by wortmannin (**Figure 6G**). In summary, SNHG12 promotes GC cell proliferation in a manner dependent on AKT pathway activation.

### SNHG12 Promotes GC Proliferation *In Vivo*

**Figure 7:**
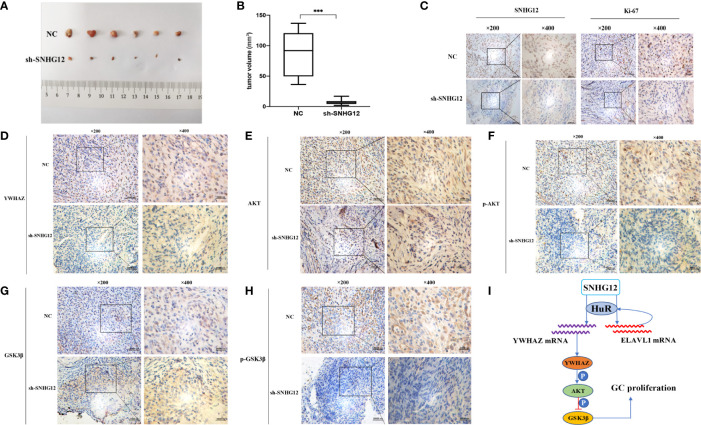
SNHG12 promotes GC proliferation *in vivo*. **(A, B)** Comparison of tumor formation between the NC groups and sh-SNHG12 groups. **(C)** SNHG12 and Ki-67 levels between the NC groups and sh-SNHG12 groups by ISH and IHC. **(D–H)** Expression of YWHAZ, AKT, p-AKT, GSK-3β, and p-GSK-3β proteins in the NC and sh-SNHG12 groups by IHC. **(I)** Schematic illustration of the mechanism underlying SNHG12 regulation of GC proliferation. Significant results were presented as ***P < 0.001. Magnification ×200, magnification ×400, scale bar 20,000 nm.

To further validate the tumor-formation potential of SNHG12 *in vivo*, MGC-803 cells stably transfected with sh-SNHG12 or empty vector were inoculated into nude mice. After 1 month, tumors in the sh-SNHG12 groups were found to be much smaller in size than those in the NC groups (**Figure 7A**). Tumor volume in the NC groups was significantly higher than that in the sh-SNHG12 groups (**Figure 7B**). ISH analysis of SNHG12 and IHC analysis of Ki67 levels in tumor tissues showed that tumors in the NC groups had higher densities than those in the sh-SNHG12 groups (**Figure 7C**). Furthermore, we performed IHC assays to measure the levels of YWHAZ, AKT, p-AKT, GSK-3β, and p-GSK-3β proteins in the NC and sh-SNHG12 groups. The results showed that the sh-SNHG12 groups exhibited lower protein levels of YWHAZ, p-AKT, and p-GSK-3β than the NC groups, while the expression of AKT and GSK-3β showed no difference (**Figures 7D–H**). The above data indicated that SNHG12 indeed promotes GC proliferation.

## Discussion

Abundant evidence illustrates that lncRNAs usually exhibit aberrant expression in various tumors and serve as vital modulators of biological processes, including cell proliferation, migration, epithelial-mesenchymal transition (EMT) and so on, which are meaningful to cancer diagnosis and therapy (20, 21). The oncogenic role of SNHG12 has been verified in recent studies, but the mechanisms underlying its role in GC are unclear. In this study, we confirmed by bioinformatics analysis and qRT-PCR assays that SNHG12 expression was upregulated in GC tissues and cells, and we also validated that SNHG12 was mainly distributed in the cytoplasm, suggesting that SNHG12 plays a role at the post-transcriptional level. Functional assays including CCK-8, colony formation assays and EdU assays illustrated that SNHG12 upregulation promotes GC cell proliferation.

To further clarify the molecular mechanisms of SNHG12 in GC proliferation, we investigated the relationship between SNHG12 and HuR, an established tumor-associated RBP. qRT-PCR showed that SNHG12 positively regulated the expression of HuR at the RNA and protein levels, while HuR could not regulate the expression of SNHG12. Furthermore, RIP assays verified that SNHG12 binds to HuR and RNA stability assays demonstrated that the SNHG12-HuR complex stabilized ELAVL1 mRNA. This newly formed complex with ELAVL1 formed a loop. Many lncRNAs can bind to HuR to stabilize mRNAs. LncRNA RMST can enhance DNMT3 expression through interaction with HuR (22); LINC00707 promotes GC proliferation and metastasis by interacting with HuR (23). Nevertheless, this is the first report that SNHG12 can regulate the expression of HuR, and that the SNHG12-HuR complex could enhance the stability of ELAVL1 mRNA. HuR has emerged as an attractive drug target for cancer therapy (24), and our works verified that SNHG12 could target HuR, thus regulating many mRNAs associated with cancer progression, which is meaningful and helpful for the development of drugs targeting HuR.

Based on previous reports and bioinformatic analysis, we hypothesized that YWHAZ is a target mRNA positively correlated with SNHG12 and HuR. The RPIseq online tool predicted that YWHAZ was highly likely to interact with HuR, and this hypothesis was supported by RIP assays. RNA stability assays demonstrated that SNHG12 and HuR regulated the stability of YWHAZ. Although previous studies reported that SNHG12 or YWHAZ could bind to HuR in other cancer types, this study is the first report that SNHG12 regulates the stability of YWHAZ by binding to HuR. Subsequently, we demonstrated that YWHAZ is highly expressed in GC cell lines and tissues by qRT-PCR and that high YWHAZ expression is related to poor survival. Cell proliferation-associated assays and WB assays demonstrated that YWHAZ promoted GC cell proliferation *via* the AKT/GSK-3β pathway. YWHAZ is of great significance in the diagnosis and treatment of various types of tumors (17). Our studies demonstrated the direct relationships among SNHG12, HuR and YWHAZ, which provides new evidence for improving tumor therapy and diagnosis.

AKT is the central node of many signaling pathways and modulates many downstream proteins involved in cellular survival, proliferation, and migration (25). It has been demonstrated that lncRNAs regulate AKT activity in direct or indirect ways (6). Previous studies reported that SNHG12 could activate the AKT pathway (26), but further elucidation is required. We found that YWHAZ could interact and phosphorylate AKT; thus, we hypothesized that SNHG12 activates the AKT pathway *via* YWHAZ. In this study, WB assays verified that AKT, p-AKT and YWHAZ expression was decreased in the sh-SNHG12 groups compared to the normal control groups. Rescue assays further demonstrated that SNHG12 promoted GC cell proliferation *via* the YWHAZ/AKT/GSK-3β axis and that this process was dependent on the AKT signaling pathways. Furthermore, according to previous studies, YWHAZ, PI3K, AKT and β-catenin can form a protein complex to stabilize β-catenin (27); YWHAZ interacts and stabilizes β-catenin by decreasing its ubiquitination degradation (19). In addition, GSK-3β can increase the degradation of β-catenin by forming a complex with APC. Axin, which upon phosphorylation by AKT, is inhibited (28, 29). Based on the above findings, we hypothesized that SNHG12 can stabilize β-catenin and increase its expression. Nevertheless, this hypothesis needs further investigations.

## Conclusion

SNHG12 functions as an oncogene in GC development and can be a biomarker for predicting prognosis. In this study, we show that SNHG12 binds to HuR to target ELAVL1 and YWHAZ, both of which are established tumor progression-related genes, and promotes GC cell proliferation *via* the YWHAZ/AKT/GSK-3β axis (**Figure 7I**).

## Data Availability Statement

The raw data supporting the conclusions of this article will be made available by the authors, without undue reservation.

## Ethics Statement

The animal study was reviewed and approved by the Ethics Committee of Ruijin Hospital affiliated to Shanghai Jiao Tong University School of Medicine.

## Author Contributions

CL and ZZ designed and funded the study. TZ and MB performed the experiments. TZ analyzed the data and made the figures. JL provided experimental material support. TZ and MB wrote the paper. ZW, YZ, JL and CL revised the paper. All authors contributed to the article and approved the submitted version.

## Funding

This study was supported by The Cross-Institutes Research Fund of Shanghai Jiao Tong University (No. YG2017MS58).

## Conflict of Interest

The authors declare that the research was conducted in the absence of any commercial or financial relationships that could be construed as a potential conflict of interest.

## Abbreviations

lncRNA, long non-coding Ribonucleic Acid; SNHG12, Small Nucleolar RNA Host Gene 12; GC, Gastric Cancer; HuR, Human Antigen R; ELAVL1, ELAV like RNA binding protein 1; YWHAZ, Tyrosine 3-monooxygenase/tryptophan5-monooxygenase activation protein; 14-3-3 protein zeta; PCR, Polymerase Chain Reaction; qRT-PCR, Reverse Transcription- quantitative Polymerase Chain Reaction; shRNA, short hairpin Ribonucleic Acid; EdU, Ethynyl deoxyuridine; FISH, Fluorescence *in situ* Hybridization; ISH, *In situ* Hybridization; RIP, RNA-Binding Immunoprecipitation; CCK-8, Cell counting Kit-8; IHC, Immunohistochemistry; IP, Immunoprecipitation.
