# Supplementary material for: Long Noncoding RNA SNHG12 Promotes Gastric Cancer Proliferation by Binding to HuR and Stabilizing YWHAZ Expression Through the AKT/GSK-3β Pathway
Source: Front Oncol. 2021 Jun 14;11:645832. doi: 10.3389/fonc.2021.645832 (PMC8236831; doi:10.3389/fonc.2021.645832)
Supplement: Supplementary file 2 [file Table_1.docx]

**Primers used for qRT-PCR：**

| GAPDH F | CCCATCACCATCTTCCAGGAG |
| --- | --- |
| GAPDH R | CTTCTCCATGGTGGTGAAGACG |
| YWHAZ F | TTTCTCCTTCCCCTTCTTCCG |
| YWHAZ R | GCCAGTTTGGCCTTCTGAAC |
| SNHG12 F | TCTGGTGATCGAGGACTTCC |
| SNHG12 R | ACCTCCTCAGTATCACACACT |
| ELAVL1 F | AAGCACCCGAAGACGGTTAG |
| ELAVL1 R | GCTGCGAAAAGCACATGGAA |

**Primers list used in RIP assays:**

| RIP-SNHG12-1F | CCTTCTCTCGCTTCGGACTG |
| --- | --- |
| RIP-SNHG12-1R | TTACCCCGGAAGTCCTCGAT |
| RIP-SNHG12-2F | ACAGGCGGATAAAACGGTCC |
| RIP-SNHG12-2R | AGTACGCCGGGATCTCTGTA |
| RIP-SNHG12-3F | GGGCCTACAGGATGACTGAC |
| RIP-SNHG12-3R | CAACCAGGTCCCCTGCATTT |
| RIP-SNHG12-4F | GGCTGACAGGCGGATAAAAC |
| RIP-SNHG12-4R | GTACGCCGGGATCTCTGTAG |
| RIP-YWHAZ-1F | ACTCCCGTTTCCGAGCCATA |
| RIP-YWHAZ-1R | CTCCAAGATGACCTACGGGC |
| RIP-YWHAZ-2F | AAAGGTCTAGGACCGCTTCC |
| RIP-YWHAZ-2R | CCAAGATGACCTACGGGCTC |
| RIP-YWHAZ-3F | CTCTCGATTGGAACGCCTCC |
| RIP-YWHAZ-3R | ACTGGATGTTCTGCTGGCTC |
| RIP-YWHAZ-4F | CCATCACTCAGCCACACTCA |
| RIP-YWHAZ-4R | GGCCTTCTGAACCAGCTCAT |

**si-RNAs used in this research:**

|  | sense（5'-3'） | antisense（5'-3'） |
| --- | --- | --- |
| si-HuR | GAACGAAUUUGAUCGUCAATT | UUGACGAUCAAAUUCGUUCTT |
| si-YWHAZ | GATGACATGGCAGCCTGCATGAAGT |  |
